# Microglial Phenotyping in Neurodegenerative Disease Brains: Identification of Reactive Microglia with an Antibody to Variant of CD105/Endoglin

**DOI:** 10.3390/cells8070766

**Published:** 2019-07-23

**Authors:** Douglas G. Walker, Lih-Fen Lue, Thomas G. Beach, Ikuo Tooyama

**Affiliations:** 1 Molecular Neuroscience Research Center, Shiga University of Medical Science, Otsu 520-2192, Japan; 2 School of Life Sciences and Neurodegenerative Disease Research Center, Arizona State University, Tempe, AZ 85287, USA; 3 Civin Laboratory of Neuropathology, Banner Sun Health Research Institute, Sun City, AZ 85351, USA

**Keywords:** neuroinflammation, neuropathology, transforming growth factor, activation, microglia, immunohistochemistry, human

## Abstract

Inflammation is considered a key pathological process in neurodegenerative diseases, including Alzheimer’s disease (AD) and Parkinson’s disease (PD), but there are still mechanisms not understood. In the brain, most microglia are performing essential homeostatic functions, but can also respond to pathogenic stimuli by producing harmful pro-inflammatory cytokines or free radicals. Distinguishing between damaging and homeostatic microglia in human diseased brain tissues is a challenge. This report describes findings using a monoclonal antibody to CD105/Endoglin (R&D Systems MAB1097) that identifies subtypes of activated microglia. CD105/Endoglin is a co-receptor for transforming growth factor beta (TGFβ) receptor that antagonizes TGFβ signaling. CD105/Endoglin is a marker for vascular endothelial cells, but was originally identified as a marker for activated macrophages. This antibody did not identify endothelial cells in brain sections, only microglia-like cells. In this study, we examined with this antibody tissue section from middle temporal gyrus derived from human brains from normal control subjects with low-plaque pathology, high-plaque pathology, and AD cases, and also substantia nigra samples from control and PD cases, in conjunction with antibodies to markers of pathology and microglia. In low-plaque pathology cases, CD105-positive microglia were mostly absent, but noticeably increased with increasing pathology. CD105-positive cells strongly colocalized with amyloid-beta plaques, but not phosphorylated tau positive tangles. In substantia nigra, strong microglial CD105 staining was observed in microglia associated with degenerating dopaminergic neurons and neuromelanin. In PD cases with few surviving dopaminergic neurons, this staining had decreased. By Western blot, this antibody identified polypeptide bands of 70 kDa in brain samples, and samples from microglia, macrophages, and brain endothelial cells. In comparison with other tested CD105 antibodies, this antibody did not recognize the glycosylated forms of CD105 on Western blots. Overall, the data indicate that this antibody and this marker could have utility for subtyping of microglia in pathologically-involved tissue.

## 1. Introduction

Neuroinflammation has been considered a prominent feature of Alzheimer’s disease (AD) and Parkinson’s disease (PD) pathology since the identification of strongly immunoreactive major histocompatibility class II (MHCII)-positive microglia associated with AD and PD pathological structures [1,2,3]. These early studies established the hypothesis that microglial inflammatory responses to extracellular amyloid-β protein (Aβ) plaques and neurofibrillary tangles, the hallmarks of AD pathology, or to degenerating dopaminergic neurons or α-synuclein structures in PD might be accelerating neurodegeneration through the excessive production of inflammatory cytokines and enzymes. If this was correct, then anti-inflammatory agents, such as non-steroidal anti-inflammatory drugs, should be effective in slowing disease progression. Despite findings from human neuropathology and many experimental animal studies, clinical trials of anti-inflammatories have generally not shown protective effects for AD subjects [4]. In diseases with accumulations of aggregated and toxic proteins, the manipulation of microglial phenotypes using non-specific pharmaceutical agents might affect their ability to remove toxic proteins, thus accelerating and not preventing neurodegeneration. The concept that all increased microglial activity could be harmful now appears incorrect as microglia are continuing to perform many essential homeostatic and phagocytic functions even in brains affected by AD or PD [5].

Increased understanding of neuroinflammation processes in AD and PD brains will come from further phenotyping of microglia for different functional markers. Increased expression of MHCII in microglia in AD cortical or hippocampal gray matter has been consistently observed, but the specificity, mechanisms, and consequences of this is still unclear. Ionized calcium binding adaptor molecule-1 (IBA-1), the other widely-used microglia marker, does not discriminate between phenotypes of microglia in human brains. A range of other markers, including CD68, a lysosomal-associated membrane protein associated with phagocytosis [6], CD32 and CD64, immunoglobulin Fc receptors [7,8], Toll-like receptors (TLR)-2, 3 [9] and 4, ferritin [10], CD163 [11], as well as TREM-2 and CD33 [12,13], microglial genes with genetic associations to AD, are among others studied. In this report, we describe the patterns of expression of microglia identified with a particular antibody to CD105/endoglin. CD105 has been used as a marker for endothelial cell activation, but was initially characterized by its expression in activated human macrophages [14,15,16].

CD105 is a type-I transmembrane glycoprotein that exists functionally as a homodimer. It is a co-receptor for transforming growth factor receptor and can modulate a range of cellular responses by endothelial cells or macrophages to transforming growth factor beta (TGFβ) proteins [17]. Due to the possession of an RGD tripeptide repeat, CD105 also has integrin-binding cell adhesion properties [14]. Modulation of TGFβ signaling appears to be a key property of CD105. Complete loss of CD105 is embryonically lethal in mice, but CD105 null-endothelial cells prepared from these mice showed higher proliferation rates and enhanced anti-proliferative responses to TGFβ through increased expression of TGFβ receptor and suppressed Smad1 activation [18]. CD105 expression by monocyte/macrophage cells has not been widely studied compared to its vascular functions, but increased expression occurs during the differentiation of macrophages from monocytes [16]. Recently, it was shown that mice with specific myeloid-cell knockout of CD105 had impaired immune responses with increased development of spontaneous infections, reduced phagocytosis, and altered responses to TGFβ [19].

There have been a number of studies concerning TGFβ in AD, but with conflicting results. TGFβ1 immunoreactivity was observed in Aβ plaques and neurofibrillary tangles in AD hippocampus [20,21], TGFβ2 in astrocytes, microglia, plaques, and tangles [21], while TGFβ1 can protect cultured neurons from Aβ peptide-induced damage [22,23]. A three-fold increase in TGFβ2 protein was measured in AD brains compared to controls [24]. In addition, TGFβ can promote microglial phagocytosis of Aβ [25] and downregulate pro-inflammatory activation in microglia induced by the endotoxin lipopolysaccharide [26]. Overexpression of TGFβ1 in experimental models can promote amyloidogenesis and cerebrovascular degeneration [27,28], while increased TGFβ1 activation of Smad2/3 signaling maintains microglia in a quiescent state [29]. Serum levels of TGFβ1 were significantly reduced in AD cases compared to aged controls, while those of soluble CD105/endoglin were increased [30]. TGFβ signaling in AD has been discussed in several key review articles with the consensus being that enhanced TGFβ signaling would be protective rather than detrimental [31,32,33].

This report describes the expression of a variant of CD105 by microglia in human brains. This feature was identified with a monoclonal antibody that recognized a subtype of microglia in fixed tissue sections but surprisingly not cerebrovascular endothelial cells. Although not recognizing brain endothelial cells in brain tissue, the antibody recognized one form of CD105 by Western blot in microglia, macrophages, brain samples, and endothelial cells. Based on previous findings of CD105 expression by macrophages and our characterization of this antibody, we suggest that this antibody is recognizing an epitope of CD105 in microglia. These findings might provide a new reagent for subtyping microglia in human pathological brains.

## 2. Materials and Methods

### 2.1. Human Brain Tissue Samples and Diagnoses Criteria

All human brain tissue samples used in this study were obtained from the Banner Sun Health Research Institute Brain and Body Donation Program, Sun City, Arizona, USA. The operations of the Brain and Body Donation Program have received continuous approval of different Institutional Review Boards (IRB). Current operations have been reviewed by Western IRB (Puyallup, WA, USA). Tissue studies carried out in the USA were considered non-human subject research under exemption 4 (C.F.R 46.101). Tissue studies carried out in Japan were approved by Shiga University of Medical Science Ethical Committee (Certificate no. 29-114). The summary of demographic details of cases used in this study are in Table 1. The cases from which middle temporal gyrus (MTG) tissue were obtained in this study were separated into set 1 and set 2. Set 1 was used for discovery studies using sections from cases donated from 1998–2010, while set 2 consisted of more recent cases collected from 2011 to 2015 and used to validate these findings. Another reason for considering these cases separately was the fact that the fixation method of brains was changed in 2007 from using freshly-prepared phosphate-buffered 4% paraformaldehyde to using commercially-prepared 10% neutral-buffered formalin. In both situations, the brain tissues were fixed for 48 hours. Tissues from three hippocampus cases were also examined for comparison. Substantia nigra samples were obtained from cases diagnosed as PD or not having disease based on clinico-pathological diagnosis. All of the substantia nigra samples used had been fixed in 4% paraformaldehyde. The substantia nigra samples from non-PD control cases had certain amounts of AD plaque and tangle pathology, but not dementia (Table 1, set 3).

All donated brains received full neuropathological diagnosis including a reference to pre-mortem clinical history of each case. Consensus clinical and neuropathological criteria were used to diagnose AD, Dementia with Lewy bodies (DLB), or Parkinson’s disease (PD) in donated cases [34,35]. To assess the severity of AD pathology in each case, tissue sections from five brain regions (entorhinal cortex, hippocampus, frontal cortex, temporal cortex, and parietal cortex) were stained with thioflavinS, Gallyas and Campbell-Switzer histological stains, and assessed semi-quantitatively for the density of neurofibrillary tangles and amyloid plaques. These methods of assessing pathological load are carried out by the neuropathology department of the Banner Sun Health Research Institute Brain and Body donation program on each donated brain as part of the diagnostic procedures, and have been extensively described over the years in their publications [36] (reviewed in Reference [37]). In brief, for each case, each brain region was ranked on a scale of 0–3 based on 0 being no plaques or tangles, 1 being few plaques or tangles, 2 being moderate numbers of plaques and tangles, and 3 being numerous plaques and tangles. By combining the measures across these five brain regions, assessment of total AD pathology can be ranked on an ordinal scale of 0–15 for plaques and tangles [36]. The two sets of cases were classified into low-plaque non-demented (LPND)(plaque score < 6), high-plaque non-demented (HPND)(plaque score 6–14), and AD with dementia (plaque score > 12). The severity of Lewy body pathology as a score of 0–40 was assessed in 10 different brain regions according to the Unified Staging Scheme for Lewy body disorders for cases from which substantia nigra sections were obtained [38].

Apolipoprotein E genotypes were determined for most cases using a polymerase chain reaction (PCR)/restriction endonuclease fragment polymorphism employing DNA extracted from cerebellum to discriminate between APOE2, APOE3, and APOE4 alleles [39]. Data on two cases in set 2 were unavailable. Results in Table 1 are presented as number of APOE4 alleles out of total numbers of APOE alleles identified in each group.

### 2.2. Immunohistochemistry

Paraformaldehyde or formalin-fixed tissue sections from temporal cortex (middle temporal gyrus), and paraformaldehyde-fixed tissue sections from hippocampus and substantia nigra were used for localization of CD105-positive cells identified with antibody MAB1097, and for colocalization with Aβ peptide and phosphorylated tau, and markers of microglia (IBA-1, HLA-DR, CD45, and P2RY12) according to our previously published procedures [9,13]. Additional staining was also carried out with other CD105 antibodies. Antibodies used in this study are listed in Table 2. Localization of bound antibody was visualized using avidin-biotin horseradish peroxidase enzyme complex (ABC-Vector Laboratories, Burlingame, CA, USA) histochemistry and nickel ammonium sulfate-enhanced diaminobenzidine as substrate to produce a purple reaction product. A second antibody was detected using the same procedure, but with diaminobenzidine without nickel ammonium sulfate as substrate to produce a brown reaction product. To demonstrate CD105-immunoreactivity in vasculature with Abcam antibody AB221675, antigen retrieval was required. For this, sections were incubated in 10 mM EDTA (pH 8.0) at 80 °C for 30 min prior to processing for immunohistochemistry.

Dual-color fluorescent confocal immunohistochemistry was carried out to verify cellular co-localization of CD105/MAB1097 expressing cells with certain other antigenic markers, as described previously [9]. Tissue sections were incubated with optimal dilutions of both antibodies at room temperature overnight with shaking. After three washes (10 min each) in phosphate-buffered saline with 0.3% TritonX100 (PBST), sections were incubated with optimal concentrations of fluorescent-labeled secondary antibodies. Bound primary antibodies were detected with Alexa 488-donkey anti-goat IgG, Alexa 568-donkey anti-rabbit or anti-mouse IgG, or Alexa 647-donkey anti mouse IgG. Sections were counterstained with Sudan Black (1% solution in 70% ethanol for 10 minutes) to quench tissue auto-fluorescence, and with DAPI (Thermo Fisher, Waltham, MA, USA) to reveal nuclei. Sections were imaged using an Olympus FV400 confocal microscope and system software.

### 2.3. Verification of Antibody Specificity

#### Absorption Tests

CD105 antibodies from R&D Systems (Minneapolis, MN, USA) (MAB1097) or Abcam (Cambridge, MA, USA.) (AB221675) were mixed for 18 hours with recombinant CD105 protein (1097-EN, R&D Systems), which consists of amino acids 26–586 of human CD105 expressed in mouse myeloma cells. This protein is glycosylated and was the immunizing peptide for production of antibody MAB1097. Antibody and protein were mixed in a ratio of 1:5 at 100-times concentration of final dilution in incubation buffer containing 0.1% bovine serum albumin as blocking agent. A parallel sample of antibody was treated without CD105 protein in blocking buffer. After dilution to working concentrations, analyses were carried out for immunohistochemistry and Western blot with these peptide-absorbed or control-absorbed antibodies.

### 2.4. Western Blot

Detergent soluble extracts from brain samples were prepared by sonicating each sample in 5 volumes of RIPA buffer (Thermo Fisher Scientific; 20 mM Tris-HCl, pH 7.5. 150 mM NaCl, 1% Triton X100, 1% sodium deoxycholate, and 0.1% sodium dodecyl sulfate) supplemented with protease and phosphatase inhibitors (Thermo Fisher). A similar procedure was used to extract proteins from cell pellets of microglia, brain-derived endothelial cells, THP-1-derived macrophages, and hCMEC/D3 brain endothelial cells. Total protein concentration of each sample was determined using a MicroBCA assay kit with bovine serum albumin as the standard. Two different gel electrophoresis methods were used in this study. For initial studies, samples were separated through 4–16% Bis-Tris NUPAGE gels (Thermo Fisher) using MOPS separation buffer, transferred to nitrocellulose and processed for detection as described (Figure 2 panels a and b and Figure 8) [9,13]. In recent studies, protein analyses, including those to assess deglycosylation and immunoprecipitations, were carried out using 4–20% Tris-glycine precast gels (Nacalai Inc, Kyoto, Japan) and Tris-glycine separation buffer (Figure 2, panels c, d and e) Proteins were transferred to nitrocellulose and processed for detection using the same procedures [9,13]. The molecular weight markers used for the NUPAGE gels were different from those used with Tris-glycine gels. Results were captured using a Fluorochem Q imaging system (Protein Simple, San Jose, CA, USA) or a ImageQuant LAS 4000 system (GE Life Sciences, Chicago, IL, USA), and band intensities measured using Image Studio Lite software (LI-COR, Lincoln, NE, USA). After initial detection, all blots (except the immunoprecipitated samples) were reprobed with an HRP-conjugated antibody to β-actin (Abcam, Cambridge, MA, USA), and reimaged for normalization purposes.

### 2.5. Deglycosylation

Protein extracts from human brains were treated with the enzyme Peptide:N-glycosidase (PNGase) F (New England Biolabs, Ipswich, MA, USA.) to remove N-linked oligosaccharides from glycoproteins. Separate protein extracts from MTG of 2 AD cases (20 μg protein/sample) were treated with denaturation buffer for 10 min at 100 °C, cooled, and then mixed with PNGase buffer and enzyme for 1 hour at 37 °C. Matching protein samples were denatured, mixed with PNGase buffer, but the enzyme was omitted for use as controls. At the end of the treatment period, samples were mixed with SDS gel buffer and dithiothreitol (DTT), and separated through 4–20% SDS polyacrylamide gels for Western blotting. To provide materials to test three different CD105 antibodies, each treated sample was divided into thirds, and separated under identical conditions for analyses.

To test the effect of deglycosylation treatment on fixed-tissue sections, they were first treated using the above-described antigen retrieval method. The deglycosylation denaturation conditions described for protein extracts could not be used for fixed tissue sections. After antigen retrieval, sections were cut into smaller pieces, and were then incubated in PNGase F buffer (with and without enzyme) for 1 hour at 37 °C. Smaller tissue sections were used so that they could be immersed in enzyme buffer and enzyme. Treated tissue sections were then processed for immunohistochemistry.

### 2.6. Immunoprecipitation

Immunoprecipitation was carried out using protein A-coupled magnetic beads (G-Biosciences, St. Louis, MO, USA) conjugated with test antibodies. In brief, protein A-beads (10 μL) were conjugated with 3 μg of antibody according to the suppliers’ instructions. As protein targets, 200 μg of protein extract from MTG of two separate AD cases and 200 μg of extract of differentiated THP-1 macrophage cells were used. Samples were mixed with antibody-conjugated beads for 18 hours (4 °C with rotation), washed three times with RIPA buffer by absorption to a magnetic tube stand, and then eluted into SDS sample buffer without DTT reducing agent by incubation at 80 °C. The omission of reducing agent and lower denaturation temperature was used to reduce the eluted immunoglobulin molecules from being denatured to molecular sizes that interfere with detection of target proteins. Samples were separated through SDS polyacrylamide gels and transferred to nitrocellulose membranes. Samples immunoprecipitated with Abcam CD105 antibody (AB221675) and Thermo Fisher CD105 antibody (PA5-32303) were detected with CD105 antibody (MAB1097) using the above-described methods. Previous experiments have shown that the mouse monoclonal antibody CD105/MAB1097 was not effective for immunoprecipitation so the reverse analyses were not attempted.

### 2.7. Cell Culture Studies

Human brain microglia and endothelial cells were prepared from frontal cortex from 3 different donor cases for this study following our published procedures [9,13]. For these experiments, the microglia and endothelial cells were derived from a PD case, a non-demented control case, and an AD case. This information was not available at the time of cellular isolation and experimentation. After 10–14 days in culture, cells were replated into wells at 10^5^ cells/well in 12-well plates prior to stimulation. For these experiments, microglia were unstimulated or treated with aggregated Aβ42 (2 μM), polyinosinic:polycytidylic acid (poly IC) (25 μg/mL), or a mixture of both agents at these concentrations [9]. After 24 hours of treatment, cells were lysed in RIPA buffer, as described above and analyzed by Western blot. Brain endothelial cells used in these experiments were not treated with stimulating agents.

The method used to prepare aggregated Aβ42 for activation of microglia has remained unchanged through multiple publications [9,13,40,41,42,43]. In brief, synthetic Aβ42 (hydrochloride salt–CPC Scientific, Sunnyvale, CA, USA) was dissolved in 5 mM sodium hydroxide, and then diluted with 10 × PBS to final concentration of 500 μM (based on protein assay of freshly dissolved Aβ42). The peptide was incubated at 37 °C for 18 h with occasional vortexing to promote aggregation. Aggregated Aβ42 was stored in aliquots in liquid nitrogen. This method produces a mixture of thioflavin S-positive fibrils and oligomers.

For use as cellular material for antibody validation studies, since human microglia protein extracts were not available for studies carried out in Japan, as a substitute, macrophage-like cells produced from the THP-1 monocytic cell line were used. THP-1 monocytes (TIB-202) obtained from the American Type Culture Collection (Manassas, VA, USA), were cultivated in suspension culture using RPMI media (Nacalai-Tesque, Kyoto, Japan) supplemented with 10% fetal bovine serum (FBS), and differentiated into adherent macrophage-like cells by treatment with 10 nM phorbol myristate acetate (PMA–Sigma-Aldrich, St. Louis, MO, USA) for 3 days in RPMI with 1% FBS. As human brain-derived endothelial cell protein extracts also were not available for studies carried out in Japan, the brain endothelial cell line hCMEC/D3 (Millipore-Merck, St. Louis, MO, USA) was used as a substitute. These cells were grown in endothelial cell media (EBM-2 with supplements, Lonzo Biologics, Portsmouth, NH, USA) on collagen-coated culture surfaces.

### 2.8. Data Analysis

Western blot data were analyzed by one-way Analysis of Variance (ANOVA) with a Newman-Keuls post-hoc test for significance between paired groups. Significant differences were assumed if P values of less than 0.05 were obtained. Statistical analyses were carried out using Graphpad Prism Version 7 software (La Jolla, CA, USA.).

## 3. Results

### 3.1. Patterns of Expression of CD105 by Microglia Identified by MAB 1097

**Figure 1:**
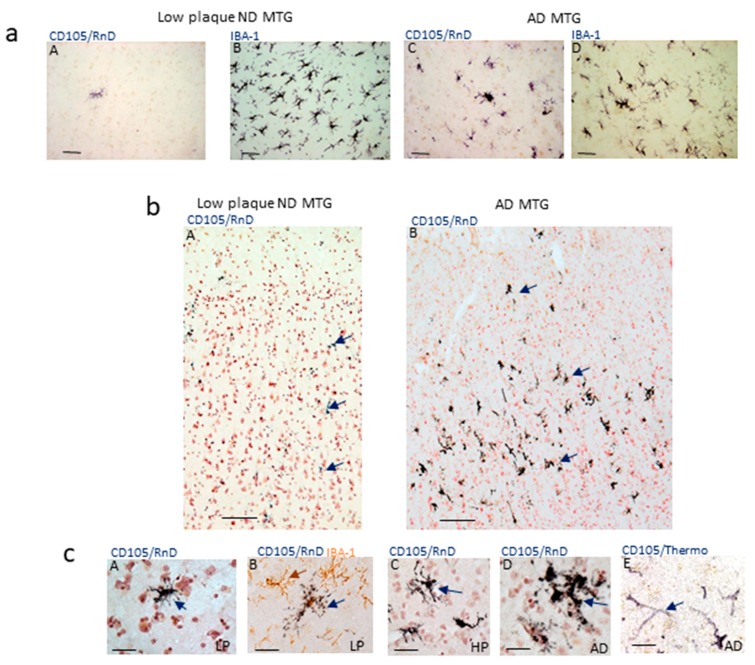
Patterns of Staining of CD105/MAB1097 in human brain sections. (**a**) Staining of parallel sections of MTG from a low-plaque ND case for CD105/MAB1097 (panel A) (CD105/RnD) and IBA-1 (panel B). Staining of parallel sections of MTG from an AD case for CD105/RnD (panel C) or IBA-1 (panel D). All images have the same magnification. Scale bars represent 50 μm. (**b**) Low magnification images showing distribution of CD105-positive cells in low-plaque ND (panel A) and AD cases (panel B). Representative positive cells indicated by purple arrows. Both images at same magnification. Scale bars represent 100 μm. (**c**) Representative image of CD105 positive cells in low-plaque ND (LP) (panel A) (purple arrow) Morphology of CD105-positive microglia compared to IBA-1-positive surrounding cells (brown arrow) in LP case (panel B). Images of CD105-positive microglia with activated morphology in HP (panel C) and AD cases (panel D) (purple arrows). (panel E) Vascular staining in AD case (purple arrow) using CD105/Thermo Fisher antibody. All images at same magnification. Scale bars represent 50 μm.

This study was initiated after testing antibody to CD105 (MAB1097-R&D Systems) to identify brain endothelial cells, but observing that this antibody identified cells with microglial morphologies. Figure 1a shows initial observations of gray matter staining with MAB1097 in middle temporal gyrus (MTG) tissue sections from an LPND case (A) and an AD case (C) in comparison to the staining pattern for the microglial marker IBA-1 in parallel sections (Figure 1a, panels B and D). Based on their morphology, it appeared that it was a subset of microglia that were immunoreactive with this antibody This observation led to this study of whether CD105/MAB1097 could be used to phenotype microglia. In all images in this publication labeled as CD105/RnD, the staining was revealed with antibody CD105/MAB1097.

The distribution of CD105-positive microglia in MTG samples had a distinct pattern. In the LPND cases (example: Figure 1b panel A), only a few positive cells were normally identified (arrows); in the AD cases there was a laminar distribution of the many intensely positive cells (Figure 1b panel B). Also, it can be seen that there was no staining of vascular endothelial cells with this antibody. The morphology of positive-staining cells varied from case to case but were consistent with microglia. In Figure 1c (panels A and B), the few CD105/RnD-positive microglia present in low-plaque (LP) cases had a non-activated morphology, while those in high-plaque (HP) or AD cases had more distinct activated morphologies (Figure 1c, panels C,D). To confirm that CD105 immunostaining of vascular endothelial cells could be identified in our series of brain sections, and the absence of vascular staining with MAB1097 was not due to tissue factors (e.g., fixation), other CD105 antibodies were employed. Using CD105/Thermo antibody (antibody PA5-32303, ThermoFisher) endothelial cells with the expected vessel morphology were positive (example of AD case (Figure 1c, panel E). This antibody did not reveal microglia staining.

### 3.2. Antibody Validation; Western Blot, Absorption, Deglycosylation and Immunoprecipitation

**Figure 2:**
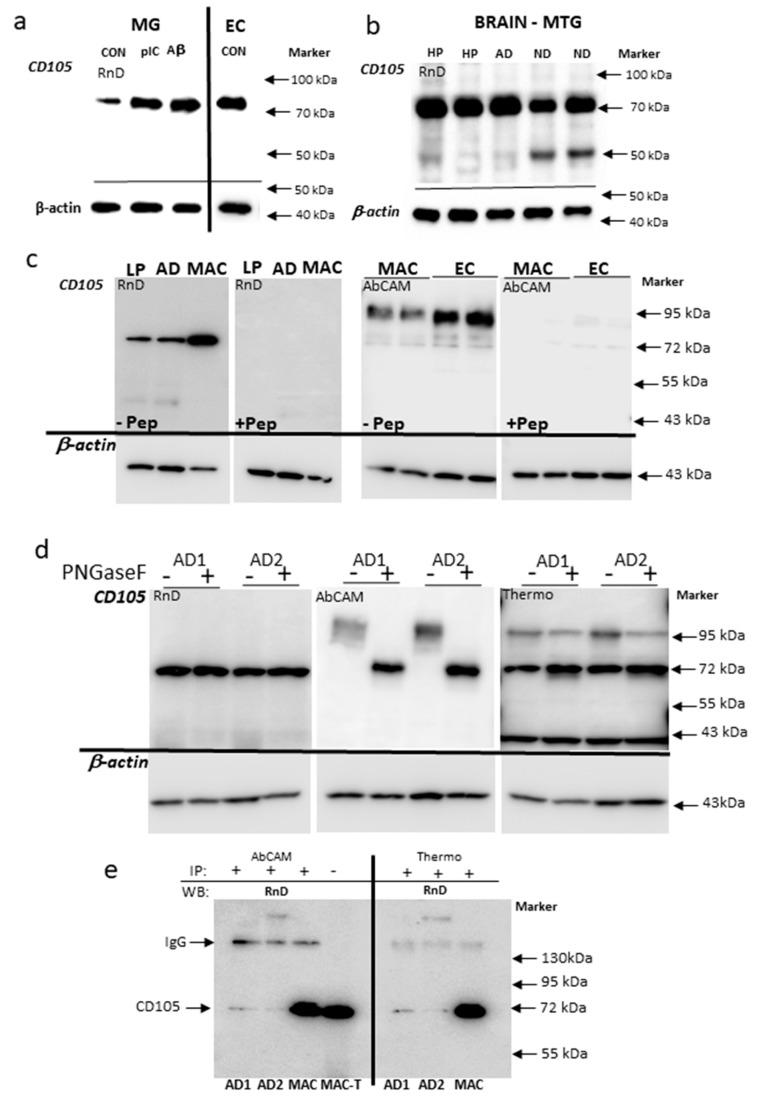
Biochemical Characterization of CD105 antibody MAB1097-detected polypeptides. (**a**) CD105 antibody MAB1097 (CD105/RnD)-identified polypeptides in protein samples of control unstimulated (CON), and polyIC (pIC) and Aβ-stimulated human brain-derived microglia (MG), and brain-derived endothelial cells (EC). (**b**) CD105 antibody MAB1097 (CD105/RnD)-identified polypeptides in protein samples of brain from middle temporal gyrus of high-plaque (HP), Alzheimer’s disease (AD) and low-plaque non-demented (ND) cases. (**c**) Antibody absorption studies. (Left hand panels): Images of Western blots containing brain MTG samples (LP, AD) and cell samples from THP-1-derived macrophages (MAC) reacted with CD105 antibody MAB1097 (RnD) preabsorbed with CD105 peptide (+Pep) or without peptide (-Pep). (Right hand panels): Images of Western blots of cell samples from THP-1-derived macrophages (MAC) or brain endothelial cell line hCMEC/D3 (EC) reacted with CD105 antibody AbCAM (AB221675) preabsorbed with CD105 peptide (+Pep) or without peptide (−Pep). (**d**) CD105 immunoreactive polypeptides after deglycosylation. Images of Western blots with CD105 antibodies MAB1097 (RnD), AB221675 (AbCAM) and PA5-32303 (Thermo) for two separate AD brain-derived protein extracts (AD1 and AD2) treated with PNGase F enzyme (+) or without enzyme (−). (**e**) Immunoprecipitation with CD105 antibodies. Images of Western blots with samples of brain (AD1 and AD2) or THP-1-derived macrophages (MAC) samples immunoprecipated with CD105 antibodies AbCAM or Thermo Fisher, and detected with CD105/RnD. MAC-T: sample of total protein of THP-1 macrophages.

To address the issue whether CD105/MAB1097 was specifically recognizing CD105 protein, we carried out antibody characterization using biochemical methods. Western blotting of protein extracts from brain-derived microglia and endothelial cells (Figure 2a) and brain (Figure 2b) showed that CD105/MAB1097 recognized a band of approximately 70 kDa in all of these samples with no detection of higher molecular weight polypeptides. To confirm the specificity of Western blot-identified bands, protein extracts of brain, macrophages, and endothelial cells were analyzed using CD105/MAB1097(RnD) peptide-absorbed antibody (Figure 2c left hand images) and CD105/AbCAM (AB221675) peptide-absorbed antibody (Figure 2c right hand images). The 70 kDa bands recognized by CD105/RnD and the 90–100 kDa bands recognized by CD105/AbCAM were noticeably reduced in intensity or absent using absorbed antibodies compared to non-peptide absorbed antibodies. The gel migration patterns suggest that MAB1097 is recognizing an unglycosylated form of CD105 and not the glycosylated forms. This was confirmed by analysis of AD brain samples treated with the deglycosylation enzyme PNGase F, in comparison with non-enzyme treated samples (Figure 2d).

PNGase F-treated brain samples when identified with CD105/RnD antibody had the same molecular weight of 70 kDa as the non-enzyme treated samples. By comparison, the same samples probed with CD105/AbCAM antibody had higher molecular weight-immunoreactive bands, which were reduced to a molecular size corresponding to 70 kDa when PNGase F-treated. A more complex pattern was revealed when samples were probed with the CD105/Thermo antibody that recognized higher molecular bands (around 95 kDa) as well as those at 70 kDa. In the PNGase F treated samples, the higher molecular weight bands were diminished in intensity, while the 70 kDa bands were increased (Figure 2d right hand image).

Immunoprecipitation analyses were also carried out to determine whether polypeptides precipitated by CD105 antibodies CD105/AbCAM and CD105/Thermo could be detected on Western blots by CD105/RnD antibody. Figure 2e shows that proteins precipitated from brain samples (AD1 and AD2) and a THP macrophage sample (MAC) by either of these antibodies could be identified with CD105/RnD at a molecular weight of 70 kDa.

### 3.3. Antibody validation: Immunohistochemistry Absorption and Deglycosylation Studies

**Figure 3:**
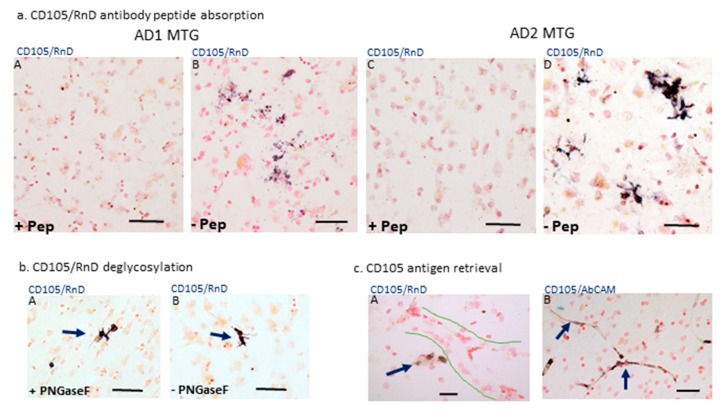
Absorption controls for CD105 staining of microglia and antibody characterization. (**a**) Antibody absorption. (panels A–D). Staining of sections from AD cases (AD1) (A and B) or AD2 with CD105 antibody preabsorbed with immunizing peptide (+Pep) compared to staining of matched sections with CD105 antibody non-absorbed (−Pep). All images are at same magnification: scale bars represent 50 μm. (**b**) Deglycosylation. Tissue section (AD case) was treated with PNGase F in solution (+PNGase F) (panel A) or underwent control treatment without enzyme (−PNGase F) (panel B), and then reacted with CD105/RnD antibody. Images are at same magnification: scale bars represent 50 μm. (**c**) Antigen retrieval. Tissue sections from an AD case underwent antigen retrieval procedure prior to immunocytochemistry with antibodies CD105/RnD (panel A) or CD105/AbCAM (panel B). The position of an unstained large vessel is outlined (panel A). Vessel staining indicated with purple arrows (panel B). Scale bars represent 50 μm.

To address whether MAB1097 was recognizing a form of CD105, we performed absorption studies using the CD105 peptide (R&D systems, 1097-EN), the immunizing peptide used for production of this monoclonal antibody. As strong CD105-positive staining was more abundant in the AD cases, we stained sections from three different AD cases with CD105-peptide preabsorbed antibody or control-absorbed antibody. From each case, adjacent sections were used. In one case (Figure 3a panel B) positive staining was punctate, while in the other it was stronger and revealed the morphology of positive-staining cells (Figure 3a panel D). However, for all sections examined, staining with peptide-absorbed antibody showed almost complete removal of immunoreactivity. 

Pretreatment of sections with PNGase F did not result in any changes in staining patterns with MAB1097 (Figure 3b, panels A and B). We also tested CD105 antibody (CD105/AbCAM). This antibody only produced strong vessel-immunoreactivity if tissue sections underwent antigen retrieval procedure (Figure 3c, panel B), but did not reveal the microglial staining pattern of MAB1097. On account of this observation on antigen retrieval, we also tested whether staining with MAB1097 might reveal endothelial cells if tissue sections also underwent antigen retrieval. However, this pretreatment did not produce this outcome (Figure 3c, panel A). Other controls included deletion of primary antibody and incubation of sections with antibody CD105/MAB10972 (R&D Systems). This latter antibody is a different IgG1 clone compared to MAB1097, but was prepared with the same recombinant protein. However, it produced no microglial or vascular staining by immunohistochemistry.

### 3.4. Expression of Microglial CD105 in Different Brain Regions and in Samples with Increasing AD-Type Pathology

**Figure 4:**
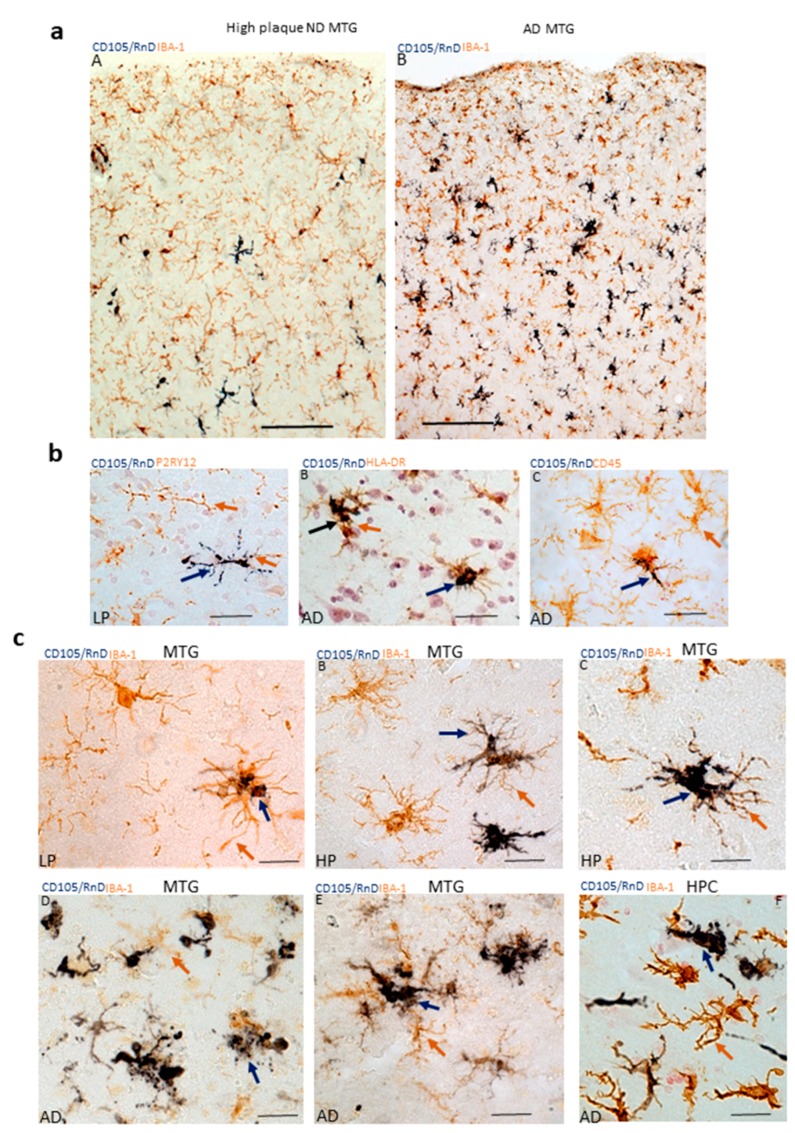
Images demonstrating colocalization of CD105/MAB1097 with microglial markers (**a**) panels A, B Low magnification images of high-plaque (HP) case (panel A) and AD case (panel B) showing staining with CD105/MAB1097 (purple arrows) and IBA-1 (brown arrows) in MTG sections. Both images at same magnification: scale bars represent 100 μm. (**b**) (panel A) CD105 microglia (purple arrows) with ramified morphology stained with antibody to P2RY12 (brown arrow) in low-plaque (LP) case. (panel B) CD105 microglia (purple arrows) and HLA-DR (brown arrow) in AD case. (panel C) CD105 microglia (purple arrow) and CD45 (brown arrow) in AD case. All sections were from MTG. Images at same magnification: scale bars represent 50 μm. (**c**) (panel A–E). Patterns of staining of CD105 (purple) and IBA-1 (brown) in MTG sections of cases with progressively increasing pathology from LP to AD. (panel F). CD105-positive microglia in hippocampus (HPC) of AD case. Images at same magnification: scale bars represent 50 μm.

The next stage was to confirm CD105/MAB1097 staining was identifying microglia, and to characterize the microglia being identified. This was first carried out by dual-color enzyme immunohistochemistry using antibodies to microglia/macrophage markers IBA-1, HLA-DR, CD45, and P2RY12. A selection of images from the large series of cases examined are shown in Figure 4.

The intensity of CD105 immunoreactivity appeared to vary with the degree of activation and morphology. It is noticeable that strong CD105-immunoreactivity occupied the cell body, but was more limited in processes that still had brown IBA-1-immunoreactivity (Figure 4c, panels B and C). In AD cases (D-F), where accumulations of microglia around pathological structures are a feature, a range of microglial morphologies were noticeable.

**Figure 5:**
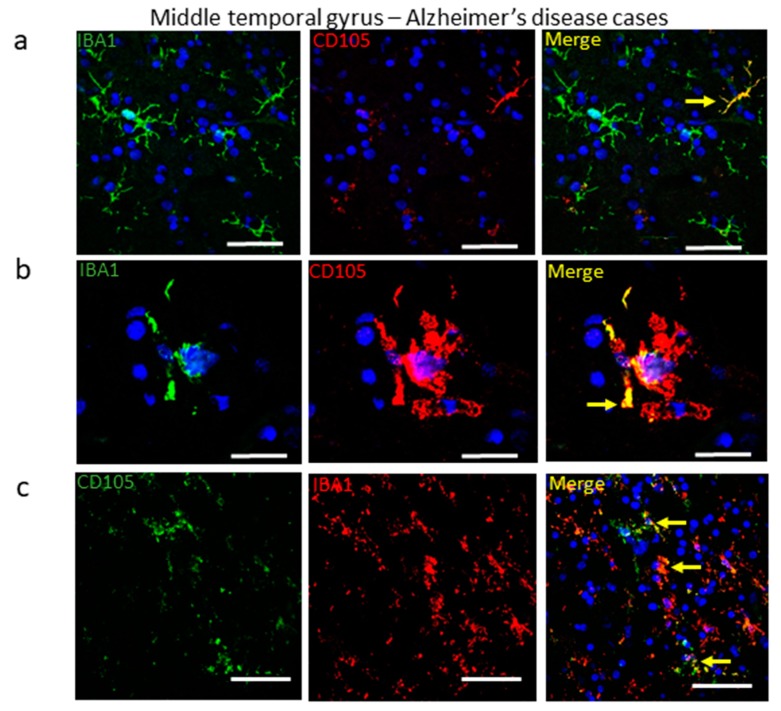
Different patterns of colocalization of CD105/MAB1097 and IBA-1 in microglia in AD cases by confocal microscopy. (**a**) IBA-1-positive (green) microglia; CD105-positive immunoreactive cells (red); Merged image showing colocalized CD105 and IBA-1 (yellow). Blue identifies nuclei revealed by staining with DAPI. Scale bars represent 50 μm. (**b**) Higher magnification image of a single cell with activated morphology. IBA-1-positive (green) microglia, strongly staining CD105-positive microglia with activated morphology (red). Merged image showing colocalized signals (yellow) within cell. Scale bars represent 25 μm. (**c**) Images to show different amounts of CD105 expression in microglia in an AD case. CD105 immunoreactivity (green), IBA-1 immunoreactivity (red), and merged image (yellow). Scale bars represent 50 μm.

In the images shown in Figure 4, colocalization of CD105/MAB1097 with microglial antigenic markers was noticeable. These tissue studies were carried out using peroxidase-based immunohistochemistry with two-color diaminobenzidine substrate detection methods. This method provides a stable product that can be extensively studied without deterioration, and allows clearer identification of the distribution of microglia in different cortical regions. This method does have limitations for definitively demonstrating antigenic-colocalization within the same cell. For this reason, we also performed multiple-color laser-confocal microscopy on selected samples to confirm that CD105/MAB1097-immunoreactive cells were microglia. This was carried out using IBA-1, a marker of all microglia, as the second antibody. As numbers of CD105-positive microglia were less abundant in LPND and HPND cases compared to AD cases, confocal colocalization with IBA-1 was only examined in MTG sections of AD cases. Figure 5a show images of microglia (yellow–Merge) demonstrating colocalization of CD105 (red) with IBA-1 (green). Figure 5b shows how a strongly-stained CD105-positive microglia with a highly-activated morphology (red) occupies the cell body with the IBA-1-staining (green) being more restricted, but where colocalization (yellow) can be seen (Figure 5b Merge). Figure 5c reverses the detection colors, but in this severe AD case, there are varying degrees of colocalization of CD105 immunoreactivity (green) with IBA-1 (red) as yellow in all cells (Figure 5c).

### 3.5. Expression of Microglial CD105 in Substantia Nigra of Control and Parkinson’s Disease Cases

**Figure 6:**
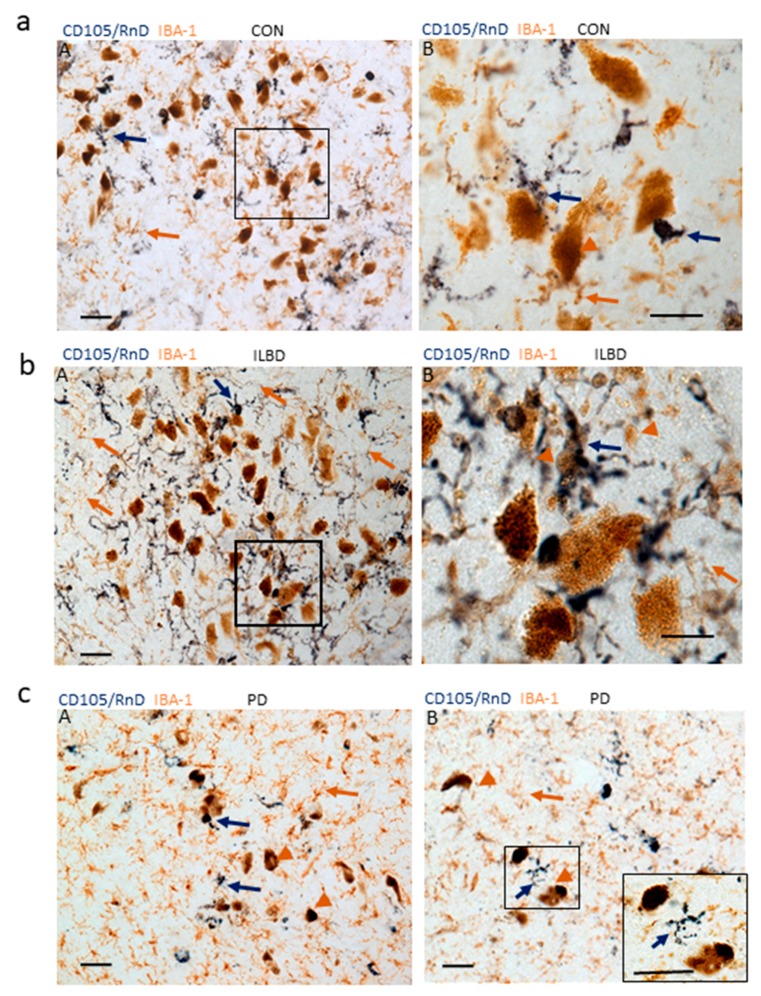
Features of CD105/MAB1097-positive microglia in substantia nigra. (**a**) Control (non-PD case). (panel A) Association of CD105-positive microglia (purple arrows) and IBA-1 positive microglia (brown arrows) with substantia nigra dopaminergic neuromelanin-containing neurons. (panel B) Higher magnification showing interaction of CD105-positive microglia (purple) with degenerating neuromelanin containing dopaminergic neurons. The area imaged in B) is indicated by boxed area in A). Scale bars represent 50 μm. (**b**) ILBD case). (panel A) Association of CD105-positive microglia (purple arrows) and IBA-1-positive microglia (brown arrows) with substantia nigra dopaminergic neuromelanin-containing neurons showing greater numbers of CD105/RnD positive microglia. (panel B) Higher magnification showing greater interaction of CD105-positive microglia (purple arrows) with degenerating neuromelanin-containing dopaminergic neurons and free neuromelanin. Examples of some neuromelanin structures indicated by brown arrowheads. The area of image in B) is indicated by boxed area on A). Scale bars represent 50 υm. (**c**) PD cases. Sparse numbers of CD105-positive microglia (purple arrows) associated with surviving dopaminergic neurons in two separate PD cases panel A and B. The inset in panel B represents the boxed area at higher magnification. Scale bars represent 50 μm).

Microglial activation has also been studied in relation to mechanisms that lead to the loss of dopaminergic neurons in the substantia nigra. Substantia nigra brain-sections from subjects with Parkinson’s disease (PD) frequently have few surviving dopaminergic neurons, but can have significant amounts of free neuromelanin released from dying or dead dopaminergic neurons. We have observed PD substantia nigra samples with few surviving dopaminergic neurons where the pathology had “burnt out” and there are few remaining activated microglia, and also PD substantia nigra samples where there is evidence of large numbers of activated microglia and ongoing pathology [44,45]. A noticeable feature in this study was the numbers of CD105-positive microglia in close association with dopaminergic neurons in cases without clinical diagnosis of PD. All samples were from elderly cases with moderate amounts of plaque and tangle pathology, but no Lewy body pathology. The Lewy body scores for 5 of the 6 control cases were 0, while one case had a score of 20. This one case was considered as having incidental Lewy body disease (ILBD), a pathological precursor to PD. Figure 6 shows representative images of the distribution of CD105/MAB1097- and IBA-1-positive microglia in these cases.

There were noticeably greater numbers of CD105/MAB1097 positive microglia in most of the control cases with surviving neuromelanin-containing dopaminergic neurons compared to the PD cases with few. All control cases examined showed evidence of degenerating dopaminergic neurons with released neuromelanin granules in the surrounding neuropil (Figure 6a, panels A and B). This was even more noticeable in the non PD/ILBD (Figure 6b, panels A and B). This case showed large numbers of CD105-positive microglia surrounding and engulfing free neuromelanin (Figure 6b, panel B). By comparison, Figure 6c (panels A and B) shows representative images from two different PD cases where there were few CD105-positive microglia associated with the neuromelanin-containing dopaminergic neurons.

### 3.6. Interaction of CD105 Microglia with Pathological Structures

**Figure 7:**
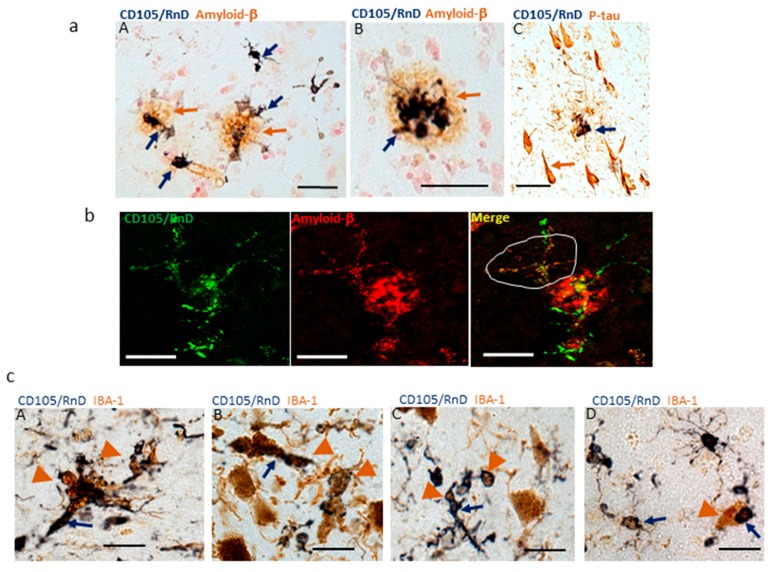
CD105/MAB1097-positive microglia associated with pathological structures. (**a**) panels A–B). Strongly CD105-positive microglia were mainly associated with Aβ plaques (panels A, B) but not with phospho-tau positive neurofibrillary tangles (C). CD105/RnD staining observed in p-tau positive neuritic plaque. Scale bars represent 50 μm. (**b**) Confocal colocalization of CD105 and Aβ. Colocalization of CD105/RnD (green) and Aβ (red) and merged image (yellow) demonstrated by confocal microscopy in AD case. The area delineated on Merge image encompasses a microglia with intracellular Aβ (yellow structures). Images are at the same magnification; scale bars represent 20 μm. (**c**) CD105-positive microglia containing neuromelanin. Panels A–D) Strongly positive CD105 positive structures (purple arrows) associated with neuromelanin particles (arrowheads) in the substantia nigra. Panel A, Control case; Panel B, ILBD case; and panel C and D, PD cases. Images are at the same magnification; scale bars represent 50 μm.

In all cases examined with Aβ plaques, particularly AD cases, the CD105-positive microglia strongly colocalized with Aβ plaques (Figure 7a, panels A and B), but not with neurofibrillary tangles (Figure 7a, panel C). In that panel, the CD105-positive staining was associated with a neuritic plaque containing Aβ protein, not with the tangles. The pattern of CD105-positive microglia (green) associated with an Aβ plaque (red) is also shown by confocal microscopy (Figure 7b). In Figure 7b–Merge, an area is outlined that corresponds to a CD105-positive microglia with colocalized Aβ immunoreactivity. This cell is not on the main plaque structure and indicates that some CD105 positive microglia have a phagocytic phenotype. This is supported by images of the substantia nigra, which continues the observations from Figure 6, but shown at higher magnification. In the Figure 7c series, CD105-positive microglia were surrounding neuromelanin granules, a feature suggestive of phagocytosis. In the following panels, it can be seen that the larger neuromelanin granules have been internalized by some of these CD105 (RnD)-positive cells. In all images (Figure 7c, panels A–D), neuromelanin can be identified as brown granules (indicated on images as arrowheads). These can be distinguished from the brown IBA-1-immunoreactive microglia.

### 3.7. In Vitro Induction of CD105 Using Human Brain Microglia

**Figure 8:**
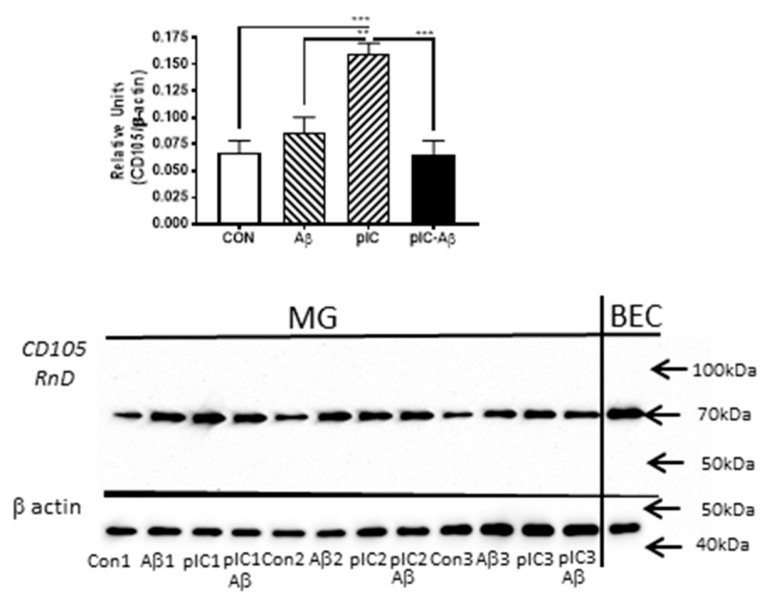
Levels of CD105–70 kDa polypeptide in in vitro-stimulated human brain microglia. Top panel: Bar chart showing mean levels of CD105 70 kDa polypeptide in stimulated human microglia. Microglia were stimulated with Aβ(42)(2 μM), poly IC (pIC) (25 μg/mL) or combination of both (pIC-Aβ) in triplicate for 24 hours and analyzed by Western blotting. Significant increase in 70 kDa in pIC-stimulated but not Aβ-stimulated microglia. Results normalized against values for β-actin. This is representative of 3-independent experiments with different microglia isolates. *** *p* < 0.001; ** *p* < 0.01. Lower panel: Western blot showing bands in treated microglia (MG). The same 70 kDa band was detected in sample of brain endothelial cells (BEC) isolated from same case. Blots were reprobed with antibody to β actin for normalization purposes.

To assess whether the induction of CD105/MAB1097-detected 70 kDa-polypeptide band corresponded with microglial activation, we used protein extracts from cultured human brain derived microglia that had been stimulated with aggregated Aβ42 (2 μM) or with 25 μg/mL of polyinosinic:polycytidylic (pIC)(TLR-3 ligand) or a mixture of both. These experiments were repeated using 3-different human brain microglia isolates with similar results. The data shown in Figure 8 represents results from cortical microglia isolated from a PD case with moderate levels of AD-type plaque and tangle pathology. Aβ42 stimulation induced CD105 70 kDa levels, though this did not reach statistical significance. By comparison, pIC treatment resulted in significant increase in CD105 70 kDa levels. Interestingly, the combination of both agents resulted in inhibition of CD105 induction. The bands being measured in microglia migrated at the same molecular weight as that present in a sample of brain-derived endothelial cells (BEC).

## 4. Discussion

This report describes immunohistochemical studies of human brain tissues from control, Alzheimer’s disease (AD) and Parkinson’s disease (PD) cases using a monoclonal antibody to CD105/endoglin that identifies types of microglia and not, as would be expected, vascular endothelial cells [17]. Detailed analyses with this antibody using absorption, Western blots, deglycosylation, immunoprecipitation, and comparison with other CD105 antibodies indicated that a variant of CD105/endoglin was being identified in microglia.

Our characterization of this antibody established that it can recognize a single polypeptide of around 70 kDa, which corresponds to the unglycosylated form of CD105, and not the higher molecular weight glycosylated forms of CD105. This was confirmed by the treatment of brain extracts with the enzyme PNGase F, which removes N-linked oligosaccharides from proteins. While the 70 kDa bands detected by CD105/MAB1097 did not change after deglycosylation, the higher molecular weight bands around 95-100 kDa detected with antibody CD105/AbCAM were reduced to 70 kDa. In addition, another CD105 antibody (CD105/Thermo) detected both higher and lower molecular weight polypeptides; the higher molecular weight bands only partially shifted to 70 kDa with deglycosylation. Overall, these results seem to confirm that the antibody that identifies microglia in tissue sections is identifying an unglycosylated form of CD105. Multiple different modifications of CD105 exist that are differentially recognized by different antibodies. The degree of glycosylation of CD105 protein can significantly affect its binding properties to form functional homodimers or bind other proteins [46]. Preabsorption of antibody with the immunizing protein, which prevented the identification of microglial cells in tissue sections and prevented identification by two different CD105 antibodies on Western blots, provides extra evidence for antibody CD105/RnD specifically recognizing a form of this protein in tissue. This was supported by the immunoprecipitation studies showing CD105/MAB1097 capable of identifying proteins precipitated by CD105/AbCAM and CD105 Thermo antibodies.

There are some features that further studies will need to address. By immunohistochemistry, MAB1097 identifies microglia, but not endothelial cells, even though the 70 kDa band can be detected by Western blots in samples of brain endothelial cells, brain, microglia, and macrophages. By contrast, the endothelial-staining antibodies did not identify microglia in tissue sections, but can identify CD105 polypeptides in brain, endothelial cells, and macrophage protein extracts by Western blots. The reason for this discrepancy is unclear. At present, one can suggest that MAB1097 could be recognizing a specific protein conformation of CD105 present in fixed microglia but not in endothelial cells. After denaturation for Western blot analysis, this form is revealed in other CD105-expressing cells. There is precedent for this: The tangle-identifying antibody Alz50 has been shown to represent a folded form of tau, but can recognize a polypeptide band of 68 kDa on gels of AD brain extracts [47,48]. Confirmation of this feature of CD105 recognized in microglia will require purification and characterization of microglia/macrophage CD105 protein, which is hindered at present due to the MAB1097 antibody not being effective for immunoprecipitation.

The experiments using in vitro-cultured microglia confirmed the in vivo observation that this form of CD105 was increased to some extent in stimulated cells. The signaling pathways of CD105 induction in microglia are not known. However, previous studies of macrophage expression of CD105/endoglin provide context for these observations [14,49]. CD105 was first identified as a marker of activated macrophages [14], as a TGFβ-binding protein, and as a modulator of TGFβ signaling in macrophages or macrophage-like cell lines [50,51]. Overexpression of CD105 reduced TGFβ cellular responses [52], but a more recent study in mice has shown that loss of macrophage CD105 expression results in reduced immune functions, including phagocytosis [19]. Expression of macrophage CD105 was triggered by their differentiation from monocytes to macrophages [19].

CD105/endoglin is also expressed by certain types of cancer and stem cells [53,54,55,56]. This study is the first to describe staining of microglia in human brains and suggests that it could be a marker for types of activated microglia in tissue. We established that CD105/MAB1097 positive cells in brain tissue were microglia based on colocalization with the microglia marker IBA-1, and also with activation markers HLA-DR and CD45. As expected in human tissue studies, there are exceptions as some microglia that were positive for CD105 in low-plaque cases had ramified resting morphologies and showed colocalization of CD105 with P2RY12, considered a marker for non-activated microglia [5] (Figure 4c). These microglia would not be considered activated. 

We observed CD105-positive microglia with Aβ plaques but not with phosphorylated tau positive neurofibrillary tangles in AD cases. The findings in substantia nigra was of strongly positive CD105-positive microglia associated with, surrounding, or even phagocytosing, neuromelanin released from damaged dopaminergic neurons. The finding of reduced numbers of CD105-positive microglia in PD substantia nigra is contrary to expectations of their being increased numbers of activated microglia in PD; however this finding is dependent on the cases being examined, and the ones we examined had severe dopaminergic cell loss. These PD cases appear to represent a “burn-out” of the PD inflammatory response. It has been demonstrated that neuromelanin has significant activation properties on microglia [57]. This result does agree with our previous finding of larger numbers of HLA-DR-positive activated microglia in elderly non-PD brain substantia nigra that correlated with levels of extracellular neuromelanin [45]. The increased numbers of CD105-positive microglia associated with degenerating dopaminergic neurons in an incidental Lewy body disease (ILBD) case is consistent with ILBD and could represent a pathological precursor stage to PD with ongoing dopaminergic neurodegeneration at a stage before PD is evident [38,44]. The noticeably fewer CD105-positive microglia associated with the degenerating neurons in PD substantia nigra would suggest that the microglial activation stimuli were no longer present.

Further studies will be needed to confirm that CD105 expressed by microglia is functional as a TGFβ signaling antagonist to determine if these observations have functional significance. The significance of TGFβ signaling has been shown to have a central role in modulating activation states and proliferation of microglia [58]. Continued exposure to TGFβ is required for microglia to develop the molecular signature of adult homeostatic microglia, and in the absence of TGFβ or TGFβ-signaling, microglia develop a pro-inflammatory profile [58]. TGFβ in brain has many anti-inflammatory and neuroprotective effects, but there appears to be increased levels of TGFβ in CSF and serum (and presumably brain) of AD and PD cases [59,60,61].

## 5. Conclusions

This study demonstrated increased expression of a variant of CD105/endoglin in a class of activated microglia in brains of AD pathology, and those with dopaminergic neuron damage in the substantia nigra. These observations were dependent on the unexpected properties of a single monoclonal antibody (R&D Systems- MAB1097) to CD105/endoglin. Biochemical characterization of proteins identified by this antibody compared to other CD105 antibodies, along with absorption studies with recombinant CD105 protein, showed that the monoclonal antibody was recognizing a polypeptide of the molecular size of unglycosylated CD105, but not the higher molecular forms of this protein. This was confirmed by biochemical deglycosylation analysis. The pattern of expression of these immunoreactive cells in relation to microglial morphology and pathological structures is consistent with CD105-expressing microglia being more activated and possibly phagocytic. At present, it is not possible to know if the antigen recognized by CD105 on microglia is a functional TGFβ binding antagonist. Further mechanistic studies are needed, but the findings in this report show that this antibody would be a useful addition to the library of antibodies that can be used to phenotype microglia in diseased human brains.

## Figures and Tables

**Table 1:** Demographic Details of Human Brain Cases Used.

**Disease State (n)**	**Age**	**Sex**	**APOE4**	**Plaques**	**Tangles**	**Braak**
*Discovery set 1: Middle temporal gyrus.*						
LPND (*n* = 11)	83.4 ± 6	7M/4F	3/22	2.8 ± 3.0	3.9 ± 2.4	I-IV
HPND (*n* = 6)	85.5 ± 5.4	3M/3F	2/12	9.7 ± 1.3	3.8 ± 1.5	II-III
AD (*n* = 7)	82.6 ± 6.5	5M/2F	7/14	14.6 ± 0.6	13.7 ± 2.8	V-VI
*Validation set 2: Middle temporal gyrus.*						
LPND (*n* = 12)	85.9 ± 8.9	6M/6F	1/22 *	1.3 ± 1.9	5.3 ± 1.9	I-IV
HPND (*n* = 12)	88 ± 8	4M/8F	3/22 *	11.3 ± 3.2	5.2 ± 2.0	I-IVI
AD (*n* = 11)	80.1 ± 6.2	6M/5F	8/22	14.4 ± 0.9	13.6 ± 1.8	V-VI
**Disease State (n)**	**Age**	**Sex**	**APOE4**	**Plaques**	**Tangles**	**LB Score**
*Set 3: Substantia nigra.*						
CON (*n* = 6))	85.7 ± 9.3	1M/5F	2/12	7.1 ± 0.6	4.8 ± 4.2	3.3 ± 8.2
HPND (*n* = 12)	86.1 ± 6	3M/3F	2/12	11.4 ± 2.4	4.0 ± 2.2	20.2 ± 2.9

Abbreviations: APOE4: No. ApoE4 alleles/Total APOE alleles; Plaques: Mean plaque score ± SEM (scale 0–15); Tangles: Mean tangle score ± SEM (scale 0–15); LPND: low-plaque non-demented; HPND: high-plaque non-demented. AD: Alzheimer’s disease; CON: not Parkinson’s disease; PD: Parkinson’s disease: Braak: Range (I-VI). LB score: Mean lewy body score ± SEM (scale 0–40) * APOE results from one case unavailable.

**Table 2:** Details of antibodies used in study.

Antibody	Supplier/Cat. No.	Species	Dilution	Application
CD105	R&D Systems (MAB1097)	Mouse	1:1000	IHC, WB
CD105	R&D Systems (MAB10972)	Mouse	1:1000	IHC, WB *
CD105	Thermo Fisher (PA5-32303)	Rabbit	1:1000	IHC, WB
CD105	Abcam (AB221675)	Rabbit	1:2000	IHC, WB
IBA-1	Wako (019-19741)	Rabbit	1:1000	IHC
CD45	Biolegend (368502)	Mouse	1:1000	IHC
P2RY12	Novus (NBP2-33870)	Rabbit	1:1000	IHC
HLA-DR	LN3-Biolegend (327002)	Mouse	1:750	IHC
Aβ	6E10-Biolegend (80314)	Mouse	1:2000	IHC
Aβ	Abcam (AB2539)	Rabbit	1:1000	IHC
Ptau	AT8-Thermo fisher (MN1020)	Mouse	1:3000	IHC

**Legend**: IHC: Immunohistochemistry. WB: Western blot. * Negative result.
